# Single versus Serial Measurements of Neuron-Specific Enolase and Prediction of Poor Neurological Outcome in Persistently Unconscious Patients after Out-Of-Hospital Cardiac Arrest – A TTM-Trial Substudy

**DOI:** 10.1371/journal.pone.0168894

**Published:** 2017-01-18

**Authors:** Sebastian Wiberg, Christian Hassager, Pascal Stammet, Matilde Winther-Jensen, Jakob Hartvig Thomsen, David Erlinge, Michael Wanscher, Niklas Nielsen, Tommaso Pellis, Anders Åneman, Hans Friberg, Jan Hovdenes, Janneke Horn, Jørn Wetterslev, John Bro-Jeppesen, Matthew P. Wise, Michael Kuiper, Tobias Cronberg, Yvan Gasche, Yvan Devaux, Jesper Kjaergaard

**Affiliations:** 1 Department of Cardiology, The Heart Centre, Rigshospitalet, Copenhagen University Hospital, Copenhagen, Denmark; 2 Department of Anaesthesia and Intensive Care, Centre Hospitalier de Luxembourg, Luxembourg, Luxembourg; 3 Department of Cardiology, Skåne University Hospital, Lund, Sweden; 4 Department of Anaesthesia and Intensive Care, Helsingborg Hospital, Helsingborg, Sweden; 5 Department of Intensive Care, Santa Maria degli Angeli, Pordenone, Italy; 6 Department of Intensive Care, Liverpool hospital, Sydney, New South Wales, Australia; 7 Department of Anaesthesia and Intensive Care, Skåne University Hospital, Lund, Sweden; 8 Department of Anaesthesia and Intensive Care, Oslo University Hospital, Rikshospitalet, Oslo, Norway; 9 Department of Intensive Care, Academic Medical Center, Amsterdam, The Netherlands; 10 Copenhagen Trial Unit, Centre of Clinical Intervention Research, Rigshospitalet, Copenhagen, Denmark; 11 Department of Intensive Care, University Hospital of Wales, Cardiff, United Kingdom; 12 Department of Intensive Care, Leeuwarden Medical Center, Leeuwarden, The Netherlands; 13 Lund University, Skane University Hospital, Department of Clinical Sciences, Neurology, Lund, Sweden; 14 Department of Intensive Care, Geneva University Hospital, Geneva, Switzerland; 15 Cardiovascular Research Unit, Luxembourg Institute of Health, Luxembourg, Luxembourg; Versailles Hospital – site André Mignot, FRANCE

## Abstract

**Background:**

Prediction of neurological outcome is a crucial part of post cardiac arrest care and prediction in patients remaining unconscious and/or sedated after rewarming from targeted temperature management (TTM) remains difficult. Current guidelines suggest the use of serial measurements of the biomarker neuron-specific enolase (NSE) in combination with other predictors of outcome in patients admitted after out-of-hospital cardiac arrest (OHCA). This study sought to investigate the ability of NSE to predict poor outcome in patients remaining unconscious at day three after OHCA. In addition, this study sought to investigate if serial NSE measurements add incremental prognostic information compared to a single NSE measurement at 48 hours in this population.

**Methods:**

This study is a post-hoc sub-study of the TTM trial, randomizing OHCA patients to a course of TTM at either 33°C or 36°C. Patients were included from sites participating in the TTM-trial biobank sub study. NSE was measured at 24, 48 and 72 hours after ROSC and follow-up was concluded after 180 days. The primary end point was poor neurological function or death defined by a cerebral performance category score (CPC-score) of 3 to 5.

**Results:**

A total of 685 (73%) patients participated in the study. At day three after OHCA 63 (9%) patients had died and 473 (69%) patients were not awake. In these patients, a single NSE measurement at 48 hours predicted poor outcome with an area under the receiver operating characteristics curve (AUC) of 0.83. A combination of all three NSE measurements yielded the highest discovered AUC (0.88, *p* = .0002). Easily applicable combinations of serial NSE measurements did not significantly improve prediction over a single measurement at 48 hours (AUC 0.58–0.84 versus 0.83).

**Conclusion:**

NSE is a strong predictor of poor outcome after OHCA in persistently unconscious patients undergoing TTM, and NSE is a promising surrogate marker of outcome in clinical trials. While combinations of serial NSE measurements may provide an increase in overall prognostic information, it is unclear whether actual clinical prognostication with low false-positive rates is improved by application of serial measurements in persistently unconscious patients. The findings of this study should be confirmed in another prospective cohort.

**Trial registration:**

NCT01020916

## Introduction

Approximately half of the unconscious patients admitted after out-of-hospital cardiac arrest (OHCA) will not survive to hospital discharge[1]. The primary causes of death are post-cardiac arrest (CA) circulatory shock and neurological injury[2], the latter accounting for around two-thirds of the overall mortality[3]. Thus prediction of neurological outcome is an important part of post-CA care and the biomarker neuron-specific enolase (NSE) has been suggested as a possible prognostic marker of poor neurological outcome in OHCA patients.

NSE is a dimeric glycolytic enzyme present in cells of neuroectodermic origin including neurons, and it is released into the bloodstream at a rate proportional to the degree of neuron damage[4]. However, NSE also exists in erythrocytes and platelets[5,6], and thus hemolysis results in increased serum NSE levels. In addition, the presence of small cell lung carcinoma and neuroendocrine tumors can lead to increased NSE levels. The half-life of NSE in serum is reported as 30 hours[7] and NSE has been shown to be a sensitive predictor of the magnitude of brain damage and outcome in cerebral infarction[8], subarachnoid hemorrhage[9] and diffuse axonal injury[10]. In post-CA care, different NSE cut-off values for poor outcome have been suggested, but a definite consensus has not been obtained and thus current guidelines do not support the isolated use of NSE for outcome prediction after OHCA but state that serial NSE-measurements should be a part of multimodal prognostication strategies[11,12]. The quality of evidence for this recommendation is “very low”, and how serial NSE measurements should be applied to achieve optimal prognostication remains unclear.

Cardiac arrest trials are very expensive and so surrogate markers would be very valuable since they would permit alternative primary or secondary endpoints in phase 2 trials. We have tended to use mortality but the sample sizes required are challenging and often unrealistic to achieve.

Recently, a sub study of the Targeted Temperature Management trial (TTM-trial) has suggested that NSE measured at 48 hours after return of spontaneous circulation (ROSC) and the NSE change between 24 and 48 hours were robust predictors of poor neurological outcome in a population of patients undergoing contemporary treatment after OHCA[13]. However, prognostication of outcome in the large subgroup of patients not awake at day three after OHCA remains challenging but highly clinically relevant, and the predictive value of NSE in this subgroup of patients remains unknown.

This post-hoc study sought to investigate the ability of NSE to predict poor outcome, i.e. a cerebral performance category (CPC) of 3–5 at 180 days, in patients remaining unconscious and/or sedated at day three after OHCA. In addition we explore whether combinations of serial measurements of NSE at 24, 48 and 72 hours after ROSC add incremental prognostic information compared to a single NSE measurement at 48 hours in patients not awake at day three.

## Methods

The patients in this study were included in the multi-center, randomized, parallel-group, assessor-blinded clinical Targeted Temperature Management trial (TTM-trial) comparing TTM at 33°C versus 36°C in comatose patients admitted after OHCA of a presumed cardiac origin at sites participating in the biobank sub study. All patients were enrolled between November 2010 to January 2013, and final follow-up was concluded in July 2013. The present study is based on post-hoc analyses from the TTM-trial.

The main trial showed no difference in outcome between the two TTM levels[14]. Eligible patients had a Glasgow coma score (GCS) below 8, were adults (age ≥ 18) and had sustained ROSC for more than 20 minutes. Main exclusion criteria included unwitnessed CA with an initial rhythm of asystole, sustained cardiac shock and lack of randomization after 240 minutes after ROSC[14].

The participants were centrally randomized in a 1:1 fashion to TTM at either 33°C or 36°C and stratified according to site. Pre-hospital data were systematically collected according to the Utstein guidelines[15]. All participants were sedated and mechanically ventilated, Neurological prognostication was postponed to 108 hrs after ROSC according to protocol and systematic criteria for withdrawal of treatment were predefined[14,16,17]. Biomarkers for brain damage were not analyzed until after all neurological prognostication was completed and were not used for prognostication. Local ethical committees in each of the participating countries approved the study and written informed consent was obtained or waived according to national legislation on all participants. The TTM-trial was registered on www.clinicaltrials.gov (identification no. NCT01020916), and was conducted in adherence with the Declaration of Helsinki. The authors confirm that all on-going and related trials for this intervention are registered.

The involved ethics committees were:

Australia: Health Ethics Review Committee Protocol No X11-0150 & HREC/11/RPAH/216 –“GI-CCT886

Czeck Republic: Ethics committee of the General University Hospital of Prague, c/j 193–11 S 17.2.2011

Denmark: De Videnskabsetiske Komiteer i Region Hovedstaden, H-1-2010-059

Italy: Comitato Etico Indipendente, Hospedaliera S Maria degli Angeli Pordenone, No 9

Luxembourg: Comité National d’Ethique de Recherche CNER No 201007/05 Ver 1.0

The Netherlands: Medisch Etische Toetsingscommissie MEC 10/107 # 10.17.0921

Norway: Regional komité for medisinsk och helsefaglig forskningsetikk Sør-øst C Ref 2010/384

Sweden: Regional Ethical Review Board Lund, Protocol 2009/6 Dnr 2009/324 (TTM-Trial)

Switzerland: Comité d’Ethique de Recherche CER 10–254 (NAC 10–088)

United Kingdom: Cardiff and Vale Research Review Service, Project ID 10/AIC/4927, Research Ethics Committee for Wales: 10/MRE09/41

### TTM population

A total of 939 patients were enrolled in the TTM-trial, and no significant differences in neurological outcome or mortality were discovered between the 33°C and the 36°C group[14]. A total of 700 patients were enrolled from sites partaking in the biomarker substudy[13]. No difference in the level of NSE between the TTM groups at any time points were discovered, and therefore the present analysis is based on the pooled data of patients in the biomarker study[13]. The local investigator defined the patients’ level of consciousness at day three after admission as awake ‘yes/no’ as part of the mandatory capturing in the study case report form, and for this study ‘no’ defined the patient as being unconscious. This definition has been used previously[18]. Throughout the manuscript “persistently unconscious” refers to patients being “unconscious and/or sedated at day 3”.

### NSE measurements

Serum blood samples were drawn at 24 hours, 48 hours and 72 hours after ROSC. The blood was centrifuged, aliquoted and frozen to -80°C at the different sites and transported to the Integrated Biobank in Luxembourg for analyses. NSE analyses were performed at the clinical biology laboratory of the Centre Hospitalier de Luxembourg 6 months after follow-up was completed. The Roche hemolysis index was used to test all serum samples for hemolysis. Samples with a positive hemolysis index (≥500mg/mL of hemoglobin) were discarded. The NSE value in each sample was analyzed using the COBAS e601 line with an Electro-chemi-luminescent-immuno-assay (ECLIA) kit (Roche Diagnostics, Rotkreuz, Switzerland) yielding a measuring range of 0.05ng/mL to 370ng/ml. Samples with NSE-values > 370ng/ml were diluted[13]. At concentrations of 10.5ng/ml and 83.3ng/ml the between-run precision was 6.8% and 5.7% respectively[13].

NSE was measured at 48 hours in 611 patients (89%), and 499 patients (73%) had NSE measurements at all three time points (24, 48 and 72 hours). Only patients with valid NSE measurements were included in the analyses.

### Outcome

Poor outcome was defined as cerebral performance category (CPC) of 3–5 assessed 180 days after CA[19]. The CPC scale ranges from 1 to 5. One (1) represents full recovery or minor disability, 2 represents moderate disability but independent in activities of daily living, 3 represents severe disability, 4 represents coma or vegetative state and 5 represents death[20].

### Statistical analysis

Differences in demographic variables at baseline were analyzed using the one-way ANOVA or chi-square test as appropriate. Demographic variables were stratified according to quartiles of NSE measured 48 hours post randomization, which has been shown to be a strong predictor of poor neurological outcome in our population[13]. Continuous variables are presented as mean ± standard deviation (SD) or median with 25th and 75th percentile inter quartile range (IQR) if skewed. Categorical variables are presented as numbers and percentages. A single NSE measurement at 24 and 72 hours as well as a number of models applying serial NSE measurements were compared to a single NSE measurement at 48 hours post ROSC. The following models with potential easily definable cut-off values applying serial NSE measures were tested:

- The geometrical area under the NSE curve from 24 to 72 hours, calculated as NSE48 x 24 + ((NSE24 –NSE48) x 24/2) + NSE72 x 24 + ((NSE48 –NSE72)) x 24/2, which equals 12NSE24 + 24NSE48 + 12NSE72 (applied as a continuous variable).
- NSE at 48 hours combined with an increase in NSE (“increase” applied as a binary variable) from 24 to 48 hours.
- NSE at 48 hours combined with an increase in NSE (“increase” applied as a binary variable) from 48 to 72 hours.
- Delta NSE from 24 to 48 hours (applied as a continuous variable).
- Delta NSE from 48 to 72 hours (applied as a continuous variable).

In addition we tested three more advanced models potentially yielding the optimal prognostic information of serial NSE measures acknowledging that interpretation and clinical use of cut-off values would be more challenging in these models:

- NSE at 24 hours combined with NSE at 48 hours.
- NSE at 48 hours combined with NSE at 72 hours.
- NSE at 24 hours combined with NSE at 48 and NSE at 72 hours.

To establish the ability of the pre specified models to predict poor outcome C-statistics through logistic regression were applied to establish the area under the receiver operating characteristics curves (AUC). The AUC for different manners of measuring NSE were compared to a single measure of NSE48 using the Chi-square test for paired data using the ‘ROCCONTRAST’ statement in SAS. The comparisons were made on identical populations in order to use the Chi-square test with equal degrees of freedom. To correct the models for over fitting, bootstrapping internal validation was applied, acknowledging that the models should not be clinically applied unless they are validated in external cohorts, due to the post-hoc nature of this study. In order to adjust for confounding factors multivariable models were included and adjusted for target temperature allocation group and factors known to influence mortality after OHCA including target temperature, sex, age, BMI, bystander CPR, initial shockable rhythm, shock on admission, time to ALS, time to ROSC, lactate at admission and creatinine at admission. Shock on admission was defined as systolic blood pressure below 80 mmHg in spite of fluid loading and/or vasopressor support and/or inotropic medication and/or intra-aortic balloon pump. Finally, the positive and negative predictive values of the various NSE models were calculated at a set specificity of 95%, i.e. a false-positive rate of 5% [21] (S1 Fig).

SAS software (Enterprise Guide 5.1, SAS institute Inc., Cary, NC, USA) was used for data analyses. R software, version 3.2.2, was used for the bootstrapping internal validation.

## Results

**Fig 1:**
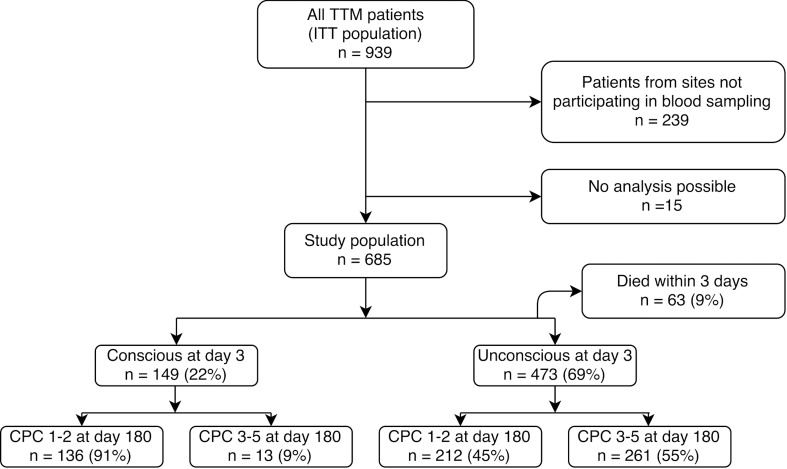
Patient inclusion. Displaying the total Targeted Temperature Management (TTM) population, Intention-To-Treat (ITT) population and outcomes by the Cerebral Performance Category (CPC) scale.

A total of 685 (98%) patients were included in the study (Fig 1). The median NSE-value at 24, 48 and 72 hours in patients treated with TTM 33°C were 24 ng/mL, 21 ng/mL and 18 ng/mL respectively (IQR 15–40 ng/mL, 13–52 ng/mL, 11–46 ng/mL) compared to 22 ng/mL, 21 ng/mL and 16 ng/mL (IQR 15–41 ng/mL, 13–67 ng/mL, 10–48 ng/mL) in patients treated with TTM 36°C. There were no significant differences between TTM groups at any of the three timepoints (*p* = 0.78, *p* = 0.95 and *p* = 0.26, respectively).

Participants were stratified according to NSE quartiles and higher NSE values were associated with several pre- and in-hospital risk factors of poor outcome (Table 1).

**Table 1:** Base line characteristics stratified by neuron-specific enolase (NSE) -quartiles in 685 included patients.

	NSE 1^st^ quartile	NSE 2^nd^ quartile	NSE 3^rd^ quartile	NSE 4^td^ quartile	*p*
NSE at 48 hours, ng/mL; min—max	2.5–13	13–21	21–59	59–782	
Distribution, n (%)	152 (25)	153 (25)	153 (25)	153 (25)	
TTM 36°C, n (%)	81 (53)	75 (49)	76 (50)	82 (54)	ns
Sex, male, n (%)	126 (83)	121 (79)	126 (82)	122 (80)	ns
Age, years, mean (SD)	60 (14)	64 (13)	65 (11)	65 (10)	**< .01**
BMI, mean (SD)	26 (3.9)	26 (4.1)	26 (4.4)	27 (5.1)	ns
CA in public, n (%)	74 (49)	84 (55)	63 (41)	43 (28)	**< .0001**
Witnessed CA, n (%)	136 (89)	140 (92)	137 (90)	136 (89)	ns
Bystander CPR, n (%)	114 (75)	119 (78)	115 (75)	98 (64)	**< .05**
Bystander defibrillation, n (%)	16 (11)	23 (15)	19 (12)	8 (5.2)	ns
Initial shockable rhythm, n (%)	138 (91)	134 (88)	134 (88)	96 (63)	**< .0001**
Time to BLS, minutes; median (25–75 percentile)	0 (0–2)	0 (0–2)	1 (0–2)	1 (0–4)	**< .05**
Time to ALS, minutes; median (25–75 percentile)	9.0 (6.0–12)	7.0 (5.0–11)	9.0 (6.0–12)	10 (7.0–15)	**< .001**
Time to ROSC, minutes; median (25–75 percentile)	19 (13–29)	20 (15–30)	27 (19–40)	37 (25–55)	**< .0001**
Shock on admission*, n (%)	7 (4.6)	9 (5.9)	31 (20)	28 (18)	**< .0001**
Arterial lactate at admission, mmol/L, mean (SD)	5.9 (4.0)	5.1 (3.4)	7.1 (4.6)	8.4 (4.8)	**< .0001**
Awake day 3, n (%)	60 (39)	56 (37)	20 (13)	1 (1)	**< .0001**

*Reversible shock that allowed for inclusion within 4 hours.

### Patients dying before day 3

Before day 3 after admission 63 (9%) of the patients had died and were thus excluded from further analysis. In this population the geometrical area under the NSE curve yielded an AUC of 0.84 and the combination of all three NSE measures yielded an AUC of 0.87 for prediction of poor outcome (*p* = .0004).

### Patients regaining consciousness before day 3

At day three after OHCA 149 (22%) patients had regained consciousness and 13 (9%) of those had a poor outcome at 180 days follow-up (Fig 1). Of the 13 conscious patients with a poor prognosis 6 had a CPC score of 3 at day 180 and 7 had died. Of the 7 conscious patients that died, only one patient died from a cerebral cause, two died from a cardiovascular cause, two from multi organ failure and two were reported as “other cause”.

NSE proved to be a poor predictor of poor outcome in patients regaining consciousness within 3 days (AUC = 0.62 for NSE measured at 48 hours).

### Patients remaining unconscious at day 3

At day three after OHCA 473 (69%) patients remained unconscious. Of these patients 261 (55%) had a poor neurological outcome at 180 days (Fig 1).

A multiple regression model with known risk factors for poor outcome resulted in an AUC of 0.79. When adding NSE measured at 48 hours to the model the AUC increased to 0.92 (additive effect: p < .0001).

Table 2 shows the AUC for different combinations of NSE values. A single NSE measurement at 24 hours was significantly inferior to a single measurement at 48 hours (AUC 0.71 versus 0.83, *p* < .0001, Table 2A). A single NSE measurement at 72 hours achieved a non-significant higher AUC than a single measurement at 48 hours (AUC 0.85 versus 0.83, *p* = .05), however this difference was no longer present after adjustment for confounding factors (AUC 0.92 versus 0.92, *p* = .42).

**Table 2:** Predictive values of neuron-specific enolase (NSE) for poor outcome after out-of-hospital cardiac arrest.

a. Univariable analyses	n	AUC	p*
NSE at 24 hours	408	0.71	< .0001
NSE at 48 hours	436	0.83	**(ref)**
NSE at 72 hours	397	0.85	.05
Highest NSE from 24 to 72 hours	473	0.81	.04
Area under the NSE-curve from 24 to 72 hours	370	0.84	.96
NSE at 48 hours combined with an NSE increase** from 24 to 48 hours	408	0.84	.72
NSE at 48 hours combined with an NSE increase** from 48 to 72 hours	397	0.84	.43
ΔNSE from 24 to 48 hours	408	0.83	.77
ΔNSE from 48 to 72 hours	397	0.58	< .0001
NSE at 24 and 48 hours	408	0.86	.02
NSE at 48 and 72 hours	397	0.85	.02
NSE at 24, 48 and 72 hours	370	0.88	.0002
b. Multivariable analyses***	n	AUC	p*
NSE at 24 hours	397	0.82	< .0001
NSE at 48 hours	381	0.92	**(ref)**
NSE at 72 hours	365	0.92	.42
Highest NSE from 24 to 72 hours	381	0.90	.01
Area under the NSE-curve from 24 to 72 hours	334	0.92	.96
NSE at 48 hours combined with an NSE increase** from 24 to 48 hours	369	0.92	.42
NSE at 48 hours combined with an NSE increase** from 48 to 72 hours	346	0.92	.42
ΔNSE from 24 to 48 hours	369	0.92	0.82
ΔNSE from 48 to 72 hours	346	0.80	< .0001
NSE at 24 and 48 hours	369	0.93	.004
NSE at 48 and 72 hours	346	0.93	.15
NSE at 24, 48 and 72 hours	334	0.94	.004

Predictive values, presented as the area under the receiver operating characteristics curves (AUC), of different NSE models for prediction of poor outcome (Cerebral Performance Category 3–5) at day 180 after out-of-hospital cardiac arrest in patients remaining unconscious at day 3 (n = 473).

*: *p*-value with single NSE at 48 hours as reference (Chi-Square) for identical populations

**: NSE increase as binary categorical variable.

***: Adjusted for target temperature, sex, age, BMI, bystander CPR, initial shockable rhythm, shock on admission, time to ALS, time to ROSC, lactate at admission, creatinine at admission.

NSE proved to be an equally robust predictor of poor outcome in patients remaining unconscious at day three after OHCA as in the total population of OHCA patients (AUC 0.83–0.88 versus 0.84–0.87), even after adjustment for confounding factors.

### Incremental information of more advanced regression models of three serial measurements of NSE in unconscious patients

The combination of three serial NSE measurements yielded a significant higher predictive value of four percentage points compared to a single NSE measurement at 48 hours (AUC 0.88 (0.85–0.92) versus 0.84 (0.80–0.88), *p* = .0002, Table 2A). After adjustment for confounding factors the increase in predictive value was two percent (AUC 0.94 versus 0.92, *p* = .004, Table 2B). The unadjusted model yielded the following beta-coefficients: NSE at 24 hours -0.0429, NSE at 48 hours 0.0331, and NSE at 72 hours 0.0746.

The use of bootstrapping internal validation on the univariable models decreased all AUCs by zero (for NSE at 24 hours), one (for other combinations) or two percentage points (for the combination of NSE at 48 hours and increasing NSE between 24 and 48 hours).

### Prediction of outcome with low false-positive rates in patients remaining unconscious

**Fig 2:**
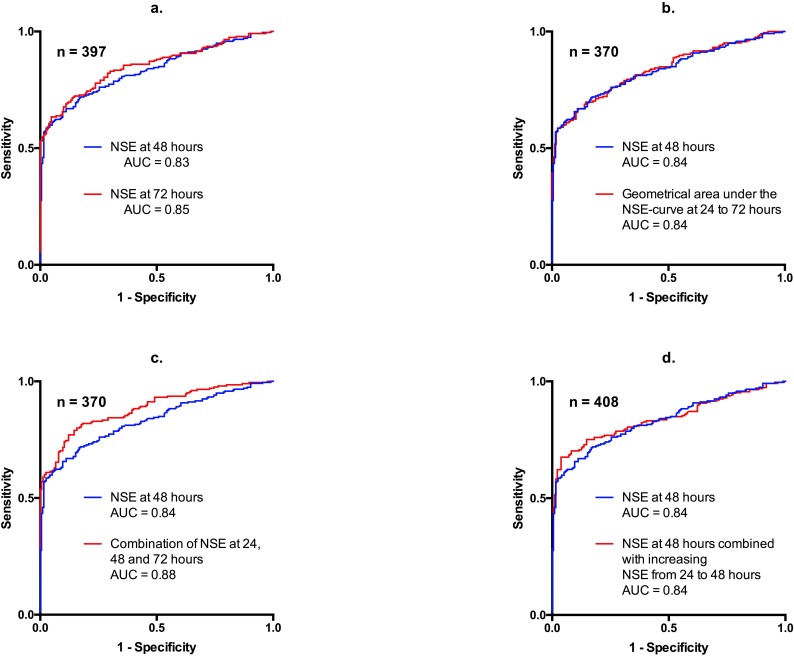
Predictive ability of neuron-specific enolase (NSE) for poor outcome after out-of-hospital cardiac arrest. Receiver Operating Characteristics plots showing the predictive ability of NSE models to predict poor outcome (CPC 3–5) at 180 days after OHCA in patients remaining unconscious at day 3 with inserts showing the enlarged plots for low false-positive rates from zero to five percent (AUCs presented for identical populations).

For equal low false-positive rates (i.e. high specificity) ROC curves showed very small differences in the corresponding sensitivity for a single NSE measurement at 48 hours versus different combinations of serial NSE measurements (Fig 2, inserts). Accordingly the confidence intervals for positive and negative predictive values of NSE measured at 48 hours were overlapping the confidence intervals of all other tested models, except for NSE measured at 24 hours and delta NSE from 48 to 72 hours, both showing inferior predictive values compared to NSE at 48h (Table 3).

**Table 3:** Predictive values of neuron-specific enolase (NSE) with a low false-positive rate.

	n	PPV*	NPV**
		% (95CI)	% (95CI)
NSE at 24 hours	439	86 (75–93)	51 (46–56)
NSE at 48 hours	436	94 (89–97)	67 (61–72)
NSE at 72 hours	425	94 (89–97)	68 (62–73)
Highest NSE from 24 to 72 hours	473	93 (88–97)	64 (58–69)
Area under the NSE-curve from 24 to 72 hours	370	94 (88–97)	66 (59–72)
NSE at 48 hours combined with an NSE increase*** from 24 to 48 hours	408	94 (88–97)	65 (59–71)
NSE at 48 hours combined with an NSE increase*** from 48 to 72 hours	397	93 (87–97)	64 (58–70)
ΔNSE from 24 to 48 hours	408	94 (89–97)	68 (62–74)
ΔNSE from 48 to 72 hours	397	90 (82–96)	56 (50–62)
NSE at 24 and 48 hours	408	94 (90–97)	71 (64–76)
NSE at 48 and 72 hours	397	94 (89–97)	67 (61–73)
NSE at 24, 48 and 72 hours	370	94 (89–97)	67 (60–73)

Positive- and negative predictive values of different NSE models, at a set specificity of 95% (equal to a false-positive rate of 5%), for prediction of poor outcome (Cerebral Performance Category 3–5) at day 180 after out-of-hospital cardiac arrest in patients remaining unconscious at day 3 (n = 473).

* PPV: Positive predictive value—the probability of a patient having a poor outcome if the NSE test is positive.

**: NPV: Negative predictive value—the probability of a patient having a good outcome if the NSE test is negative.

***: NSE increase as binary categorical variable.

## Discussion

Patients remaining unconscious after OHCA have a poor prognosis and the decision of whether to continue or withdraw life-sustaining therapy remains a significant clinical challenge. Thus a marker that with a low false-positive rate can predict poor outcome in persistently unconscious patients would be of great clinical interest, and NSE has been suggested as such a marker. This extended analysis of the TTM NSE study[13] showed that single NSE measures drawn at 48 and 72 hours have high predictive values for poor outcome in OHCA patients remaining unconscious after rewarming from TTM, even after adjustment for potential confounding factors. The predictive ability of NSE measured at 24 hours was significantly inferior in unconscious patients. In contrast, NSE proved to be a relatively weak predictor of poor neurological outcome in patients regaining consciousness within the first three days, which corresponds to the observation that only one patient regaining consciousness later died from a cerebral cause.

Current guidelines for prognostication in OHCA patients advocate using serial NSE measurements in a multimodal prognostication strategy[12], and accordingly, in persistently unconscious patients we found a significantly higher AUC when combining three NSE-measurements versus a single measurement at 48 hours in the total population. For clinical use in predicting OHCA patients’ outcome however, the key issue is not the overall predictive ability of a given model but the ability to predict poor outcome in unconscious patients with a very low false-positive rate, i.e. a very high specificity. For equal high specificities of 95%, frequently used as a reasonable degree of accuracy for diagnostic testing, the differences in sensitivity as well as positive and negative predictive values of NSE measured at 48 hours versus the combination of the three NSE measures were marginal (Fig 2, inserts, Table 3) with overlapping confidence intervals. However, clinically useful differences may still exist and these models for combining serial NSE measurements should be tested in external cohorts. In addition applying an advanced model incorporating three NSE measures may present a challenge in interpretation and in turn in determining relevant cut-off values for use in the clinical setting. Thus we evaluated simpler, more clinically applicable algorithms combining serial measurements with the intention to predict poor outcome with as high a predictive value as possible for equal low false-positive rates.

One argument supporting the use of serial measurements is to avoid false-positive results due to haemolysis, and a practical approach to this has been to use the NSE value at a single time point (ex. 48 hours) combined with an increasing/decreasing NSE trend between two time points (ex. 24 to 48 hours). In theory, haemolysis on admission will result in a decreasing NSE trend from 24 to 48 hours due to the half-life of NSE in serum. Also, on-going hypoxic brain injury will result in an increasing NSE trend from 24 to 48 hours due to persistent NSE release from dying neurons. In contrast to these theories our results showed no overall incremental prognostic value of adding the NSE trends to a single NSE value (Table 2), and the predictive values for low false-positive rates were similar in the two models (Table 3). While the confidence intervals overlap, an important explanation for this is that there are very few patients with a high NSE value and a good outcome. In addition the distribution of NSE is right skewed and thus the variance in high NSE values is relatively wide which makes significant differences difficult to discover. Accordingly, for low false-positive rates, a relative large change in NSE cut-off value is needed to change the false-positive rate by just a single percentage point (a change in false-positive rates from 0 to 5 percent results in NSE cut-off changes from 120ng/mL to 44ng/mL for NSE at 48 hours), which corresponds well with previous studies demonstrating highly varying NSE cut-off limits[22–25]. Considering the size of our present cohort a very large cohort would be needed to show any significant differences for the low false-positive rates making the establishment of useful absolute NSE cut-offs impractical due to corresponding low sensitivity. The difficulty in obtaining absolute cut-off values for NSE supports the use of multimodal prognostication including GCS motor score, somato-sensory evoked potentials etc. as suggested by the current guidelines[12]. Accordingly our multivariable analyses have shown that the combination of NSE and known risk factors increase the predictive value for poor outcome in persistently unconscious patients compared to either NSE alone or the risk factors.

Our results suggest that NSE could be useful as a surrogate marker for neurological injury in pilot trials investigating outcome after OHCA enabling the trials to include fewer patients than trials with hard outcomes. The advantage of using NSE in this setting would be that NSE, unlike most clinical markers, can be blinded for the treating clinicians and remains closely correlated to outcome.

The predictive value of NSE as assessed by c-statistics corresponded well to previously shown values[26–28]^.^ In our cohort 55% of the patients remaining unconscious at day three had a poor outcome at follow-up, which is consistent with previous studies [23,29].

The present study has some limitations, the primary being a post-hoc design with application of different mathematical and simple models of the combination of serial NSE measurements. Thus the apparent performance of our tests will tend to be optimistic due to over fitting, and our results will need external validation in another prospective cohort before any clinical implementation [30]^.^ While application of bootstrap internal validation to correct for over fitting decreased the presented AUCs by zero (for NSE at 24 hours) to two percentage points (for the combination of NSE at 48 hours and NSE increase between 24 and 48 hours) it did not alter the presented differences between the various models which emphasizes the need for external, in addition to our internal, validation. Furthermore the prognostic value of NSE in the TTM-trial has previously been reported[13] and the results of our extended analyses should thus be considered hypothesis generating only. We consider external validation to be essential before the cut-offs can be applied in clinical practice and thus we have refrained from suggesting such cut-offs based on this post-hoc analysis. The definition “unconscious at day three” include patients with a delayed awakening as well as patients being kept sedated due to various reasons, and standardized neurological prognostication was not carried out until after 4.5 days. Thus, not just patients with a delayed awakening due to neurological injury are included in the definition. The issue of clinicians withdrawing treatment selectively in a group of patients with a presumed poor prognosis may lead to a self-fulfilling prophecy in studies investigating any diagnostic method. To counter this issue all treating clinicians were blinded to the NSE values during the course of admission and until end of trial and by protocol, active intensive care was continued for a minimum of 108 hours following the arrest before standardized prognostication[19].

## Conclusion

NSE is a strong predictor of poor outcome after OHCA in persistently unconscious patients undergoing TTM, and NSE is a promising surrogate marker of outcome in clinical trials. While combinations of serial NSE measurements may provide an increase in overall prognostic information, it is unclear whether actual clinical prognostication with low false-positive rates is improved by application of serial measurements in persistently unconscious patients. The findings of this study should be confirmed in another prospective cohort.

## Supporting Information

S1 Fig STARD Checklist. (PDF)
